# Temperature-dependent macromolecular X-ray crystallography

**DOI:** 10.1107/S0907444910002702

**Published:** 2010-03-24

**Authors:** Martin Weik, Jacques-Philippe Colletier

**Affiliations:** a CEA, IBS, Laboratoire de Biophysique Moléculaire, F-38054 Grenoble, France; b CNRS, UMR5075, F-38027 Grenoble, France; c Université Joseph Fourier, F-38000 Grenoble, France

**Keywords:** temperature-dependent macromolecular crystallography

## Abstract

X-ray crystallography provides structural details of biological macromolecules. Whereas routine data are collected close to 100 K in order to mitigate radiation damage, more exotic temperature-controlled experiments in a broader temperature range from 15 K to room temperature can provide both dynamical and structural insights. Here, the dynamical behaviour of crystalline macromolecules and their surrounding solvent as a function of cryo-temperature is reviewed. Experimental strategies of kinetic crystallography are discussed that have allowed the generation and trapping of macromolecular intermediate states by combining reaction initiation in the crystalline state with appropriate temperature profiles. A particular focus is on recruiting X-ray-induced changes for reaction initiation, thus unveiling useful aspects of radiation damage, which otherwise has to be minimized in macromolecular crystallography.

## Abstract 2

The dynamical behaviour of crystalline macromolecules and their surrounding solvent as a function of cryo-temperature is reviewed.

## 1. Introduction

Macromolecular X-ray crystallography has greatly benefited from several innovations at the end of the last century, including the implementation of cryo-methods (Hope, 1990 ▶; Teng, 1990 ▶; Garman & Schneider, 1997 ▶; Garman & Owen, 2006 ▶) and the availability of brilliant X-ray beams from third-generation synchrotron sources. The timing was fortuitous, since the widespread use of the latter would not have been possible without the former. Indeed, the deleterious effects of X-ray irradiation on crystalline proteins were recognized early on (Blake & Philips, 1962 ▶) and make room-temperature experiments with modern synchrotron-based X-ray beams difficult, if not impossible. By flash-cooling a macromolecular crystal to 100 K its lifetime in the beam is increased by about 100-fold (Nave & Garman, 2005 ▶; Southworth-Davies *et al.*, 2007 ▶) because the diffusion of the radicals created by X-ray irradiation is limited in the glassy matrix of the crystal solvent. Another beneficial effect of cryocooling originates from reduced dynamic disorder. For example, about twice as many water molecules are detected at cryo-temperature compared with room temperature in protein structures determined using X-ray (Nakasako, 1999 ▶) or neutron crystallography (Blakeley *et al.*, 2004 ▶). Today, more than 90% of all macromolecular X-­ray crystal structures are determined from data collected at 100 K (Garman & Owen, 2006 ▶).

At room temperature, biological macromolecules are often active in the crystalline state (Mozzarelli & Rossi, 1996 ▶). The high solvent content of about 50% and the limited contacts that macromolecules make with each other in a crystal allow the occurrence of a large fraction of the macromolecular motions that underlie biological function in solution. We note in passing that the macromolecular crowding and solvent content in a crystal are similar to those in a biological cell. At cryo-temperatures, macromolecular motions are slowed down or cease and biological activity is impaired. One can thus turn macromolecular activity on and off by varying the temperature of the crystal between 100 K (or lower) and room temperature and thereby trap functional intermediate states that can be structurally characterized by crystallography. The corresponding temperature-controlled approach is part of a broader kinetic crystallography toolbox aimed at filming proteins in action (Bourgeois & Royant, 2005 ▶; Bourgeois & Weik, 2009 ▶).

In the following, we review some aspects of the dynamical behaviour of proteins and their aqueous environment as a function of cryo-temperature, with particular focus on crystalline proteins. We then summarize temperature-controlled protein X-ray crystallographic experiments that are aimed at (i) studying functional intermediate states, (ii) characterizing X-ray-induced damage to crystalline proteins or (iii) employing the latter to benefit the former.

## 2. Protein and solvent dynamics as a function of cryo-temperature

Over the past decade(s), intensive effort has been invested in the exploration of protein structures and rightly so. Because the delicate balance between both structural and dynamical aspects forms the basis of biomolecular function, efforts are now multiplying to uncover the ‘dynamic personalities of proteins’ (Henzler-Wildman & Kern, 2007 ▶). Studying protein motions at subzero (°C) temperatures is a valuable approach that permits the slowing down and teasing apart of the multitude of motions that otherwise occur simultaneously under physiological conditions (Parak, 2003 ▶). This dynamical complexity stems from the multidimensional energy landscape formed by the conformational substates accessible to a protein and its surrounding solvent (Frauenfelder *et al.*, 1991 ▶). Macromolecular motions lead to interconversions between substates and hence ‘bring a protein to life’. When a hydrated biological macromolecule is cooled to cryo-temperatures, anharmonic macromolecular motions cease at the so-called dynamical transition, which occurs at a temperature of between about 180 and 220 K. The dynamical transition occurs in solution as well as in powder and crystalline samples of proteins, RNA and DNA. It was first discovered by researchers using Mössbauer spectroscopy to probe haem-iron movements in myoglobin (Parak *et al.*, 1982 ▶) and has subsequently been studied by other experimental techniques including neutron scattering (Doster *et al.*, 1989 ▶; Ferrand *et al.*, 1993 ▶) and X-ray crystallography (see §[Sec sec4]4 below). In addition to its importance as a prominent feature in the low-temperature physics of biological macromolecules, the dynamical transition has been linked to the onset of biological activity (Rasmussen *et al.*, 1992 ▶; Lichtenegger *et al.*, 1999 ▶; Ostermann *et al.*, 2000 ▶). However, certain enzymes are active below the dynamical transition (Daniel *et al.*, 1998 ▶), or are at least able to undergo part of their catalytic cycle (Heyes *et al.*, 2002 ▶; Durin *et al.*, 2009 ▶).

The thin film of water around proteins, *viz.* their hydration water, is vital to the biological activity of the macromolecule. Without hydration water, proteins lack the conformational flexibility that animates their three-dimensional structures and the dynamical transition is suppressed. The solvent viscosity influences the temperature at which the protein dynamical transition occurs; the higher the viscosity, the higher the transition temperature (Beece *et al.*, 1980 ▶; Lichtenegger *et al.*, 1999 ▶). Consequently, protein dynamics are thought to be ‘slaved’ to hydration-water dynamics (Frauenfelder *et al.*, 2002 ▶). One manifestation of the tight coupling is the appearance of a glass-like transition in the hydration water at the same temperature as the dynamical transition of the protein (Wood *et al.*, 2008 ▶). In particular, the onset of water translational diffusion has been identified in molecular-dynamics simulations as the driving force behind the protein dynamical transition (Tarek & Tobias, 2002 ▶). Molecular-dynamics simulations have also suggested that water motions couple to those of the protein above the dynamical transition and that intrinsic protein motions dominate below (Vitkup *et al.*, 2000 ▶). The details of the dynamical transition, its modulation by solvent dynamics and its relation to biological activity remain hotly debated (Doster, 2008 ▶, 2009 ▶; Frauenfelder *et al.*, 2009 ▶).

## 3. Temperature-dependent behaviour of protein crystals

Cryocrystallographic experiments require that macromolecular crystals are flash-cooled in a cryogen such as liquid (63–77 K) or gaseous (typically 100 K) nitrogen, liquid propane (83–231 K) or liquid ethane (90–185 K). The goal of the rapid temperature decrease allowed by the flash-cooling is the avoidance of crystalline ice formation in the water fraction of the crystal solvent. The change in density associated with water crystallization disturbs the crystal packing and results in a deterioration in the diffraction quality. In order to avoid crystalline ice formation, the solvent needs to be vitrified to an amorphous state before the water molecules have had the time to reorient and diffuse to form a crystalline arrangement. The higher the viscosity of the solvent and the more pronounced the solvent confinement by the macromolecules, the higher the temperature of the solvent glass transition and the easier it is to avoid crystalline ice formation by vitrification. In most cases the solvent viscosity has to be raised above that of the mother liquor in which the crystal grew by the addition of penetrating cryoprotectants such as glycerol, low-molecular-weight polyethylene glycol or salts (Garman & Schneider, 1997 ▶). For some crystalline proteins, crystalline ice formation is absent during flash-cooling even without the addition of penetrating cryoprotectants. In those cases, the viscosity of the mother liquor confined in the crystal is already sufficiently high to allow vitrification by flash-cooling. Recently, it has been reported that crystalline ice formation does not occur in thaumatin crystals without penetrating cryoprotectants when the temperature is decreased from 300 to 100 K at a very slow rate (0.1 K s^−1^; Warkentin & Thorne, 2009 ▶). The potential interest of this new slow-cooling procedure in kinetic crystallography is discussed in §[Sec sec4]4. Crystalline ice formation in the absence of penetrating cryoprotectants can also be pre­vented by flash-cooling protein crystals under high pressure (200 MPa; Kim *et al.*, 2005 ▶). Once the solvent has been rendered amorphous, the protein crystal is in a metastable state at cryo-temperatures; it has ‘fallen out of thermodynamic equilibrium’. What happens when a flash-cooled protein crystal is warmed above 100 K, *viz*. the tem­perature at which most cryocrystallographic data are collected? A short summary of the behaviour of flash-cooled pure water is a prerequisite for understanding the more complex case of protein crystals.

**Figure 1:**
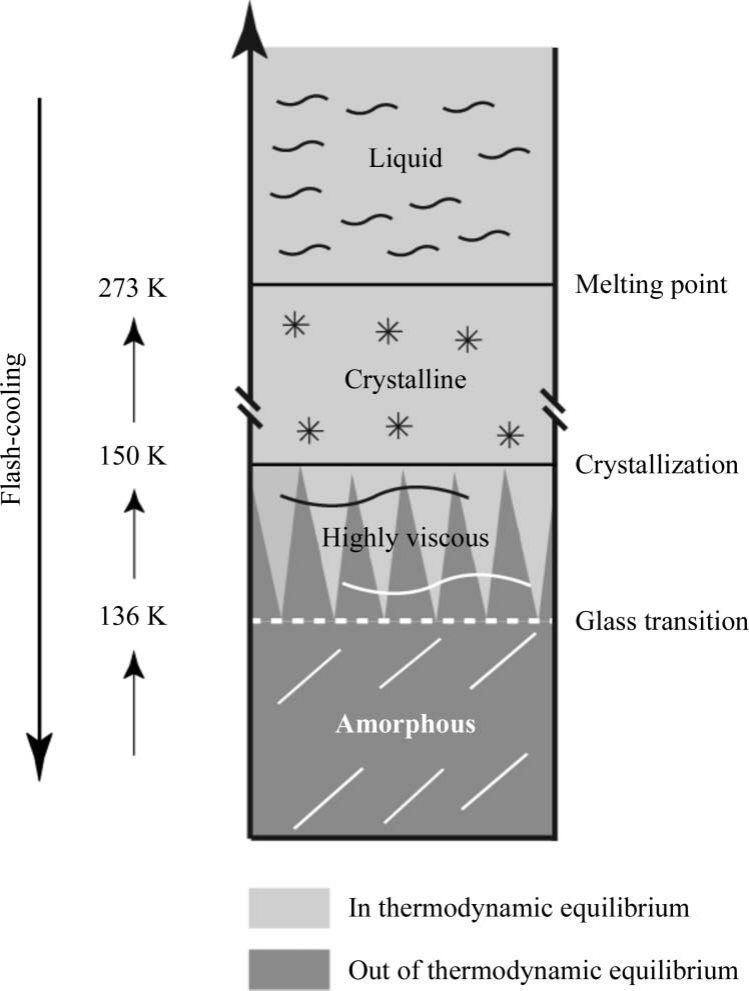
Schematic illustration of the cryo-temperature domains of flash-cooled pure bulk water at ambient pressure (after Mishima & Stanley, 1998 ▶).

Pure bulk water generally forms ordinary crystalline ice (hexagonal; I_h_) if cooled below 273 K. Under certain conditions where nucleation events are rare, water can be supercooled below 273 K. However, crystallization is inevitable when the temperature of homogeneous nucleation (235 K at atmospheric pressure) is approached (Kanno *et al.*, 1975 ▶). Crystallization can be bypassed by flash-cooling liquid water, which leads to the formation of amorphous ice (for a review, see Angell, 2004 ▶). Different forms of amorphous ice have been described. Amorphous solid water (ASW), which represents most of the water in the universe, is produced by condensing water vapour on a cold substrate. Hyper-quenched glassy water (HQGW) is formed by flash-cooling small droplets (Bruggeller & Mayer, 1980 ▶) or thin films of water (Dubochet & McDowall, 1981 ▶) at a rapid rate (10^5^–10^6^ K s^−1^). Yet another amorphous form of water, so-called high-density amorphous ice (HDA), results from the pressurization of I_h_ to 1 GPa at 77 K. Upon heating above 117 K HDA expands and transforms into so-called low-density amorphous ice (LDA; Mishima *et al.*, 1984 ▶). The transformation from HDA (1.17 g cm^−3^) to LDA (0.94 g cm^−3^) is accompanied by a 20% increase in volume (Mishima *et al.*, 1985 ▶). LDA, HQGW and ASW have similar structures and densities (Mishima & Stanley, 1998 ▶) and undergo a glass transition upon warming to 129 K (LDA) or 136 K (ASW, HQGW) (McMillan & Los, 1965 ▶; Johari *et al.*, 1987 ▶; Mayer, 1991 ▶). At the glass transition the viscosity suddenly drops and it has been proposed that amorphous water transforms into an ultraviscous liquid (Mishima & Stanley, 1998 ▶), from which it returns to thermodynamic equilibrium by crystallizing into cubic ice (I_c_) at 150 K (Mayer, 1991 ▶). Upon further warming I_c_ transforms into I_h_ at 186 K (McMillan & Los, 1965 ▶). The different forms of flash-cooled water are represented in Fig. 1 ▶. Water molecules have been reported to gain rotational freedom at the glass transition (Fisher & Devlin, 1995 ▶) and to exhibit translational diffusion just above, at 150 K (Smith & Kay, 1999 ▶). However, the existence of ultraviscous water in the temperature range between 136 and 150 K remains controversial (Kohl *et al.*, 2005 ▶; Yue & Angell, 2004 ▶) and a direct transformation from the glassy to the crystalline state at 150–160 K has been proposed (Velikov *et al.*, 2001 ▶). In any case, liquid bulk water cannot be studied experimentally at temperatures between 150 and 235 K, the so-called ‘no man’s land’ (Mishima & Stanley, 1998 ▶).

Solvent in protein crystals differs from bulk water in three ways: it contains solutes from the mother liquor and possibly from a cryoprotectant, it is in contact with macromolecular surfaces and it is confined. These three differences alter the cryo-temperature behaviour of crystal solvent with respect to that of pure water as schematized in Fig. 1 ▶. During flash-cooling, the crystal lattice (unit-cell volume) contracts by 2–7%, whereas the protein molecules only contract by 1–3% (Juers & Matthews, 2001 ▶). If the contraction of the solvent in the crystal channels and cavities does not match the crystal and protein contraction, flash-cooling reduces the crystalline order and degrades the diffraction quality by forcing a fraction of the solvent to move out of the crystal (Juers & Matthews, 2001 ▶, 2004 ▶) or into small regions scattered throughout the crystal (Kriminski *et al.*, 2002 ▶). Apart from avoiding crystalline ice formation within solvent channels, the ideal cryoprotectant at the ideal concentration (Mitchell & Garman, 1994 ▶) should contract upon flash-cooling to an extent that exactly matches the crystal and protein contractions (Juers & Matthews, 2001 ▶; Kriminski *et al.*, 2002 ▶). Juers and coworkers have measured the contractions of 26 different cryo-solutions, which ranged from 2% to 13% upon decreasing the temperature from 294 to 72 K, thus providing an experimental basis for rationally matching the thermal contraction and expansion of the different crystal components (Alcorn & Juers, 2010 ▶).

The behaviour of the solvent inside flash-cooled protein crystals upon slow warming (0.1–0.001 K s^−1^) to temperatures above 100 K will depend on its composition, the particular confinement geometry, the pressure during flash-cooling, the heating rate and the presence or absence of nonpenetrating cryoprotectants such as oils (Weik, Kryger *et al.*, 2001 ▶; Parkin & Hope, 2003 ▶; Weik, Lehnert *et al.*, 2005 ▶; Kim *et al.*, 2008 ▶, 2009 ▶). If the solvent is confined within large channels (60 Å and larger), the rate of unit-cell volume expansion as a function of temperature drastically increases as the temperature is raised above 155 K and crystalline ice rings concomitantly appear in the diffraction pattern (Weik, Kryger *et al.*, 2001 ▶). It has been concluded that the expansion results from the water fraction forming crystalline ice within the solvent channels. The unit-cell volume of crystals with channels that are 30–40 Å across has been reported to increase suddenly at 165 K, resulting either from nanocrystalline ice formation within the channels or from the glassy solvent transforming into an amorphous form of lower density (Parkin & Hope, 2003 ▶). Crystals with channels of about 20 Å showed a drastic decrease in their unit-cell volume as the temperature was raised above 190 K, accompanied by the appearance of crystalline ice rings (Weik, Schreurs *et al.*, 2005 ▶). Water is transported out of the crystal (Juers & Matthews, 2001 ▶, 2004 ▶) to crystallize at its surface, leaving a collapsing protein crystal lattice behind (Weik, Schreurs *et al.*, 2005 ▶). Solvent transport out of the crystal can be avoided in some cases by non­penetrating cryoprotectants such as oils that serve as a kinetic barrier (Juers & Matthews, 2004 ▶; Weik, Schreurs *et al.*, 2005 ▶). In the case of even smaller channels (10 Å) the solvent does not form crystalline ice and is not transported (Weik, Kryger *et al.*, 2001 ▶). In conclusion, ice formation within the crystal apparently does not occur if the channels are smaller than about 30 Å. Pure water confined in hydrophilic silica materials (Dore, 2000 ▶) has also been reported not to form crystalline ice if the channel sizes are below 28 Å (Jahnert *et al.*, 2008 ▶).

Pure water has been proposed to form an ultraviscous liquid when heated above its glass transition and prior to crystallization as outlined above. Can solvent in protein crystals also be found in an ultraviscous state at cryo-temperatures? Two pieces of evidence suggest that this is indeed the case. Firstly, the observation of solvent being transported out of a crystal with 20 Å channels at 190 K is model-free evidence that water exhibits long-range translational diffusion when it forms crystalline ice at the protein-crystal surface (Weik, Schreurs *et al.*, 2005 ▶). Similar observations have been made for flash-cooled purple membranes (Weik, Lehnert *et al.*, 2005 ▶). A second piece of evidence is provided by elegant experiments on high-pressure cryocooled protein crystals (Kim *et al.*, 2009 ▶) that contained pressure-induced HDA within their solvent channels (Kim *et al.*, 2008 ▶). Upon heating from 80 to 165 K, HDA transformed to LDA as identified by characteristic water diffuse diffraction rings of the two amorphous phases. The volume expansion of 20% accompanying the HDA to LDA transition did not cause a swelling of the solvent channels. It was concluded that water is transported to the crystal surface or into grain boundaries, thus providing evidence for its liquid-like character during the transition (Kim *et al.*, 2009 ▶). The existence of a narrow cryo-temperature window, in which the crystal solvent is liquid-like can be exploited in kinetic crystallography experiments to allow for the protein flexibility necessary to build up functional intermediate states; this is further discussed in §[Sec sec6]6.

Understanding the physical chemical properties of solvent in protein crystals in the temperature range 100–300 K also helps to rationalize the various crystal-annealing procedures that have exhibited the potential to improve the diffraction quality of flash-cooled macromolecular crystals (Yeh & Hol, 1998 ▶; Harp *et al.*, 1998 ▶; Kriminski *et al.*, 2002 ▶; Hanson *et al.*, 2003 ▶; Juers & Matthews, 2004 ▶; Weik, Schreurs *et al.*, 2005 ▶). In particular, solvent transport during annealing to room temperature has been suggested to change the cryoprotectant concentration, thereby altering the thermal contraction properties of the crystal and thus improving the diffraction quality; this implies that the cryoconditions were not fully optimized beforehand (Mitchell & Garman, 1994 ▶; Kriminski *et al.*, 2002 ▶; Juers & Matthews, 2004 ▶). Alternatively, transient liquefaction of the crystal solvent at room temperature or below could provide the necessary mobility for crystalline macromolecules to slightly rearrange, leading to a release of the lattice stress built up during flash-cooling, decreased mosaicity, reduced distribution of lattice spacings and thus improved diffraction resolution (Kriminski *et al.*, 2002 ▶; Kim *et al.*, 2009 ▶).

## 4. Protein structures at various cryo-temperatures

The structure of myoglobin determined at various temp­eratures between 220 and 300 K provided the first evidence that dynamical information could be obtained from protein crystallography (Frauenfelder *et al.*, 1979 ▶). Subsequently, several other protein structures have been studied at more temperature points and in a broader temperature range from 80 to 300 K (Singh *et al.*, 1980 ▶; Hartmann *et al.*, 1982 ▶; Parak *et al.*, 1987 ▶; Tilton *et al.*, 1992 ▶; Kurinov & Harrison, 1995 ▶; Nagata *et al.*, 1996 ▶; Teeter *et al.*, 2001 ▶; Joti *et al.*, 2002 ▶; Edayathuman­galam & Luger, 2005 ▶; Schmidt *et al.*, 2009 ▶; Kim *et al.*, 2009 ▶). Crystallographic *B* factors (Debye–Waller factors) can also provide some insights into protein dynamics. Indeed, atomic mean square displacements 〈*x* ^2^〉 extracted from *B* factors (〈*x* ^2^〉 = *B*/8π^2^) stem from both dynamic and static disorder. Extrapolating the temperature-dependence of *B* factors to 0 K provides an estimate of the static contribution. In the case of the crystalline haemprotein nitrophorin 4, the contribution of static disorder to the *B* factor averaged over all non-H main-chain atoms was 40% and 65% at room temperature and 100 K, respectively (Schmidt *et al.*, 2009 ▶). The temperature-dependence of averaged *B* factors, however, differs from protein to protein. A linear behaviour has been observed for nitrophorin 4 (Schmidt *et al.*, 2009 ▶) and myoglobin (Parak *et al.*, 1987 ▶; Chong *et al.*, 2001 ▶), whereas a biphasic behaviour of the temperature-dependence of *B* factors with a kink at a temperature between 150 and 200 K has been reported for ribonuclease A (Tilton *et al.*, 1992 ▶), crambin (Teeter *et al.*, 2001 ▶) and lysozyme (Joti *et al.*, 2002 ▶). The kink has been interpreted as a manifestation of the protein dynamical transition from harmonic to anharmonic motions and in the case of ribonuclease A it has been shown that the substrate binds to the active site above but not below the transition temperature (220 K; Rasmussen *et al.*, 1992 ▶). Likewise, the water structure at the surface of crystalline crambin decreased above the transition (200 K). Does the linear temperature-dependence of *B* factors in nitrophorin and myoglobin indicate the absence of a dynamical transition in these proteins? Joti and coworkers offered an explanation for the apparent difference in the temperature-dependence of *B* factors in different protein crystals by arguing that a dynamical transition can take place despite linearity of the *B* factors at temperatures around 200 K (Joti *et al.*, 2002 ▶). If the same set of conformational substates in the energy landscape of the crystalline protein is occupied throughout the entire temperature range studied, a dynamical transition cannot be observed by examining crystallographic *B* factors. In contrast, a transition can be observed when certain substates are depleted at lower temperature. Also, inspecting the *B* factors of individual amino acids might reveal non­linearity despite there being a linear behaviour of *B* factors averaged over the entire protein, indicating a local change in populated substates at the dynamical transition. Indeed, a reduction in the number of alternate side-chain conformations is often observed in protein structures determined at cryo-temperatures compared with structures determined at room temperature (Parak *et al.*, 1987 ▶; Dunlop *et al.*, 2005 ▶).

The population of conformational substates at cryo-temperatures strongly depends on the flash-cooling velocity. Typical values for protein crystals flash-cooled using nitrogen are of the order of 50–500 K s^−1^ and are lower if gaseous and higher if liquid nitrogen is used (Teng & Moffat, 1998 ▶; Walker *et al.*, 1998 ▶; Kriminski *et al.*, 2003 ▶). If the cold gas layer above a liquid-nitrogen surface is removed, the cooling rates can be increased to up to 15 000 K s^−1^ (Warkentin *et al.*, 2006 ▶). Halle calculated that at these cooling rates a crystalline protein falls out of equilibrium at around 200 K (Halle, 2004 ▶). Movements of side chains and water molecules are then quenched at this temperature and the protein structure determined at 100 K effectively represents that at 200 K. A way to address the degree to which protein structures are quenched during flash-cooling is to compare them with structures determined after slow cooling. Warkentin and Thorne recently showed that thaumatin crystals with solvent channels of 25–35 Å can be cooled from room temperature to 100 K at 0.1 K s^−1^ without crystalline ice formation (Warkentin & Thorne, 2009 ▶). An interesting experiment would be to determine the structures of flash-cooled and slow-cooled thaumatin crystals at various temperatures between 100 and 300 K and to compare them. If the crystalline protein indeed falls out of equilibrium at 200 K, the *B* factors and structural features such as alternate side-chain conformations and water net­works of flash-cooled and slow-cooled crystals should be similar above but different below 200 K.

## 5. The temperature-dependence of X-ray radiation damage to crystalline proteins

X-ray irradiation of macromolecular crystals during crystallo­graphic data collection leads to a decrease in diffraction quality and to specific damage to the macromolecules {see *Proceedings of the Second to the Fifth International Workshops on X-ray Damage to Crystalline Biological Samples* published in special issues of the *Journal of Synchrotron Radiation* [Vol. **9**, Part 6 (2002), Vol. **12**, Part 3 (2005), Vol. **14**, Part 1 (2007) and Vol. **16**, Part 2 (2009)]; for a review, see Ravelli & Garman, 2006 ▶}. Two types of damage are distinguished: primary and secondary. The former results from the inter­action of an X-ray photon with atoms in the sample, leading to the ejection of a highly energetic electron as a result of the photoelectric effect, which is the dominant inelastic event at the photon energies used in macromolecular crystallo­graphy (Murray *et al.*, 2005 ▶). Primary radiation damage is temperature-independent (Teng & Moffat, 2002 ▶). Secondary damage arises from the many secondary radicals created by the primary photoelectron. Radiolysis of water plays a prominent role among secondary events and leads to a variety of radicals, including hydrated electrons (e^−^_aq_), hydroxyl radicals (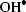), atomic hydrogen and protons.

The temperature-dependence of secondary radiation damage was the very vehicle for implementing cryo-methods in macromolecular crystallography, as pointed out in §[Sec sec1]1. At room temperature, secondary radicals are mobile and either recombine or damage the protein. At 100 K, large radicals such as 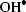 are trapped in the rigid matrix of the amorphous solvent. In contrast, electrons are mobile down to much lower temperatures and their tunnelling has been reported even at 5 K (Dick *et al.*, 1998 ▶). Whereas the benefit of cooling macromolecular crystals from room temperature to 100 K is clearly evident from the approximately 100-fold increased lifetime, the potential for reducing radiation damage by collecting data at 40 K or below is much less obvious. Only a small increase in crystal lifetime of about 25% has been reported for data collected at 15 K instead of 100 K as judged from global radiation-damage indicators (Chinte *et al.*, 2007 ▶; Meents *et al.*, 2007 ▶). With respect to that at 100 K, at 50 K specific damage to a disulfide bond has been reported to be reduced fourfold and global damage to be decreased by 50% (Meents *et al.*, 2010 ▶). On the other hand, the photoreduction of metal centres, a specific radiation-damage effect (Yano *et al.*, 2005 ▶), is reduced 30-fold at 40 K compared with 110 K as determined by X-ray absorption spectroscopy (Corbett *et al.*, 2007 ▶). The redox integrity of crystalline metalloproteins thus greatly benefits from reducing the temperature to below 100 K during data collection.

How does the radiation-sensitivity of macromolecular crystals evolve as a function of temperature above 100 K? Whereas the crystal solvent remains amorphous at temperatures below 100 K, it does not in the 100–300 K temperature range, as we have seen in §[Sec sec3]3. Above 100 K, but still below the solvent glass-transition temperature, the lifetime of radicals in the solvent (*e.g.* that of hydrated electrons) and in the protein (*e.g.* that of disulfide radicals) is temperature-independent as determined by UV–visible absorption spectroscopy per­formed online at a synchrotron beamline (McGeehan *et al.*, 2009 ▶). The sudden drop in viscosity at the solvent glass transition (which takes place at 150 K or at a higher temperature) allows an increase in the mobility of radicals that were trapped in the amorphous solvent at lower temperatures and therefore the radical lifetime decreases. As a consequence, the unit-cell volume, which increases linearly as a function of absorbed X-­ray dose at 100 K (Ravelli & McSweeney, 2000 ▶),  increases non­linearly (Weik, Ravelli *et al.*, 2001 ▶) or even decreases (Ravelli *et al.*, 2002 ▶) above the solvent glass transition and both global (Borek *et al.*, 2007 ▶) and specific (Weik, Ravelli *et al.*, 2001 ▶) radiation damage steeply increase. The cryo-temperature at which the crystal solvent turns ‘liquid-like’ and radical diffusion is increased depends on solvent composition and confinement, as discussed in §[Sec sec3]3. At the same temperature, one would expect 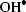 radicals to become mobile, as they do above 110 and 130 K in amorphous (M. D. Sevilla, private communication to E. Garman) and crystalline ice (Symons, 1999 ▶), respectively. However, not a single example of oxidative damage arising from the action of 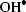 radicals has been observed to date in protein crystal structures determined at temperatures between 100 K and room temperature.

## 6. Temperature-controlled kinetic cryocrystallography to characterize protein intermediate states: exploiting dynamical transitions of solvent and protein

Dynamical transitions at cryotemperatures in proteins and in their surrounding solvent have been proposed to be linked to biological function, as outlined in §[Sec sec2]2. Temperature-controlled protein crystallography can thus be exploited to generate, trap and structurally characterize macromolecular intermediate states (Ringe & Petsko, 2003 ▶) by combining reaction triggering with appropriate temperature profiles. Together with real-time Laue diffraction close to room temperature, temperature-controlled crystallography is part of the kinetic crystallography toolbox that provides structural biologists with means to address macromolecular function *via* crystallography. Temperature-controlled kinetic crystallography either follows a trigger–cool or a cool–trigger sequence. In the former, reaction initiation is achieved at room temperature, followed by trapping of the generated intermediate state by rapidly lowering the temperature to 200 K or below. In the latter, the crystalline macromolecule is first flash-cooled and the reaction is then initiated. A reaction initiated at low temperatures, *e.g.* at 100 K, can only proceed when the protein flexibility is enhanced by raising the tem­perature, typically to above the dynamical transitions of the solvent and protein (Weik, Ravelli *et al.*, 2001 ▶; Kim *et al.*, 2009 ▶). Several ways exist of triggering a reaction, including the irradiation of endogenous or exogenous photosensitive macromolecules with UV–visible light, the diffusion of small molecules such as substrates or products and X-ray irradiation creating radicals and specific bond cleavage. Kinetic crystallo­graphy greatly benefits from complementary spectroscopy techniques, such as offline (Bourgeois *et al.*, 2002 ▶) and online (McGeehan *et al.*, 2009 ▶) UV–visible fluorescence and absorption (Pearson *et al.*, 2004 ▶; De la Mora-Rey & Wilmot, 2007 ▶), Raman (Carpentier *et al.*, 2007 ▶), EPR (Utschig *et al.*, 2008 ▶) and X-ray absorption spectroscopies (Hough *et al.*, 2008 ▶). Extensive recent reviews of kinetic crystallography exist (Petsko & Ringe, 2000 ▶; Bourgeois & Royant, 2005 ▶; Bourgeois & Weik, 2009 ▶; Hirai *et al.*, 2009 ▶) and we focus here on temperature-controlled crystallography using X-ray irradiation as a reaction trigger.

**Figure 2:**
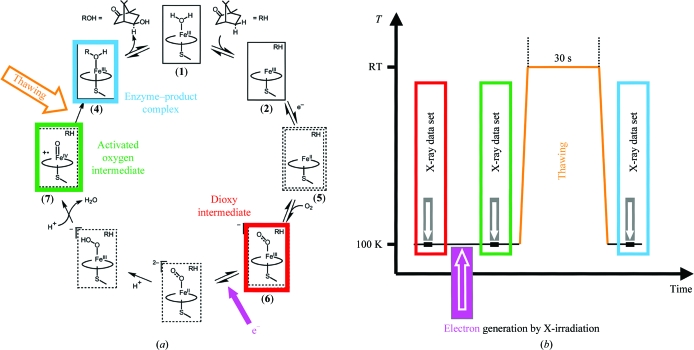
(*a*) The reaction pathway of P450cam (based on Schlichting *et al.*, 2000 ▶) and (*b*) the experimental protocol used to trap and generate the three intermediate states highlighted by coloured rectangles.

A combination of X-ray-induced electron production and temperature-controlled crystallography was used in seminal work by Schlichting and coworkers to generate, trap and characterize two of the functionally relevant intermediate states on the reaction pathway of the crystalline enzyme P450cam (Schlichting *et al.*, 2000 ▶). P450cam catalyses the hydroxylation of camphor in a 2e^−^ redox reaction. Most of the intermediates accumulate until specific triggers (two electrons, O_2_) allow the reaction to proceed a step further. The structural starting point for crystalline P450cam was its dioxy intermediate [species (**6**) in Fig. 2 ▶ *a*]. *In vivo*, the transition to the activated oxygen intermediate [species (**7**) in Fig. 2 ▶ *a*] is triggered by an electron provided by another protein (putida­redoxin) that is not present in the P450cam crystals. Instead, the electron was created by water radiolysis resulting from prolonged X-­ray irradiation of the crystal at a temperature close to 100 K. In order to maximize X-ray absorption (which is proportional to λ^3^) the wavelength was shifted from 0.9 Å (at which data were collected) to 1.5 Å during irradiation. For the transition to the enzyme–product complex [species (**4**) in Fig. 2 ▶ *a*] to occur, the protein and substrate had to gain sufficient flexibility and this was provided by a transient tem­perature rise to above the protein dynamical transition (30 s at room temperature). The experimental strategy is summarized in Fig. 2 ▶(*b*). In another example, reduction of the haem iron by synchrotron radiation followed by transient warming to room temperature was used to generate and trap intermediate states in crystalline myoglobin (Hersleth *et al.*, 2008 ▶). Research on other crystalline redox-sensitive proteins for which X-ray-induced reduction has been reported at tem­peratures close to 100 K (Berglund *et al.*, 2002 ▶; Adam *et al.*, 2004 ▶; Baxter *et al.*, 2004 ▶; Mees *et al.*, 2004 ▶; Roberts *et al.*, 2005 ▶; Echalier *et al.*, 2006 ▶; Beitlich *et al.*, 2007 ▶; Kuhnel *et al.*, 2007 ▶; Pearson *et al.*, 2007 ▶; Hough *et al.*, 2008 ▶) could benefit from temperature-controlled crystallography if structural information on intermediate states were of interest.

**Figure 3:**
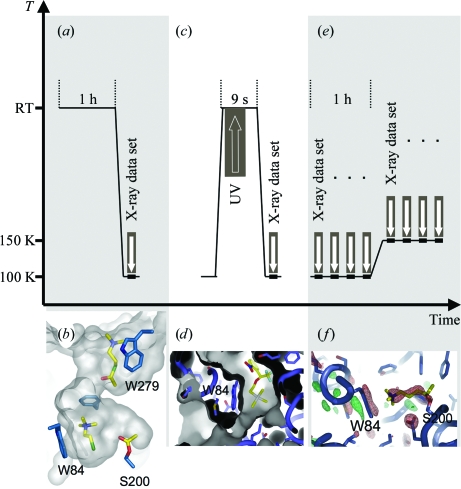
Temperature-controlled kinetic crystallography to study the structural details of substrate and product traffic in the enzyme acetylcholinesterase (adapted from Bourgeois & Weik, 2009 ▶). (*a*) Soaking AChE crystals at room temperature in an excess of the substrate acetylthiocholine led to (*b*) a steady-state population trapped at 100 K, in which a substrate, a choline and an acetyl group were located in the active-site gorge (Colletier *et al.*, 2006 ▶). (*c*) Using UV irradiation of caged choline as a reaction trigger during a brief excursion to room temperature (*d*) an intermediate state was trapped in which a small movement of Trp84 opened a channel from the active site to the solvent region (Colletier *et al.*, 2007 ▶). (*e*) When several consecutive data sets were collected at 150 K from crystals of acetylcholinesterase in complex with a nonhydrolysable substrate analogue (Colletier *et al.*, 2008 ▶), a similar movement of Trp84 (*f*) as seen in (*d*) was observed.

Temperature-controlled crystallography has also benefited mechanistic studies of acetylcholinesterase (AChE), an enzyme that plays a central role in the nervous system. AChE terminates nerve-impulse transmission at cholinergic synapses by hydrolysing the neurotransmitter acetylcholine to acetate and choline (Silman & Sussman, 2005 ▶). In order to fulfil its biological function, AChE needs to be rapid and efficient, making it difficult to explore structural and dynamical details of substrate and product traffic by crystallographic means. Up to 10 000 substrate molecules per second are hydrolysed in an active site that is located at the bottom of a deep and narrow gorge (Sussman *et al.*, 1991 ▶). Based on molecular-dynamics simulations, the existence of a ‘backdoor’ has been postulated that could transiently open near the active site and allow rapid product clearance (Gilson *et al.*, 1994 ▶). Several temperature-controlled crystallography approaches have been designed that allowed us to shed light on some aspects of the molecular traffic within AChE. In a first approach, the addition of substrate in excess to the crystalline enzyme led to the slowdown of catalysis by a phenomenon called substrate inhibition (Colletier *et al.*, 2006 ▶). As a consequence, various steady-state situations were established at room temperature and trapped by flash-cooling (Fig. 3 ▶ *a*), which permitted the visualization of different intermediates in the enzymatic reaction, *e.g.* the two products, acetyl group and choline, trapped in the active site by a substrate molecule blocking the gorge entrance (Fig. 3 ▶ *b*). Can the trapped choline product escape through a backdoor? Evidence that this can happen was provided by two further temperature-controlled experiments. In the first of these, a photolabile precursor of choline (a so-called ‘caged com­pound’) inhibited the enzyme by binding at the active site (Colletier *et al.*, 2007 ▶). Uncaging of the compound, *i.e.* the liberation of choline, was achieved by UV-laser irradiation of the crystal during a short temperature excursion of 9 s to room temperature (Fig. 3 ▶ *c*). Partial difference refinement then showed that in 20% of the crystalline enzymes a backdoor opened by small movements of Trp84 (Fig. 3 ▶ *d*). In another approach, X-ray irradiation was used to trigger a reaction. A nonhydrolysable analogue of acetylcholine that binds to the catalytic serine was radiocleaved by X-rays during the collection of a series of data sets at two temperatures (Colletier *et al.*, 2008 ▶). Four consecutive data sets were collected at 100 K and four at 150 K after having translated the crystal to a previously unexposed part (Fig. 3 ▶ *e*). The two temperature values were below and close to the solvent glass transition, respectively. By computing difference Fourier maps between sequential data sets at each temperature, different enzymatic intermediate states were trapped. At 100 K, the nonhydrolysable substrate analogue was radiocleaved and the freed pseudo-choline molecule remained trapped in the active site. At 150 K, radiolysis freed a pseudo-choline that could not be located in the active site. Instead, a pair of positive and negative difference-density peaks at Trp84 and Tyr442 indicated that these residues had moved; these movements were attributed to pseudo-choline having exited the active site through a backdoor, which therefore was suggested to be open at 150 K but not at 100 K (Fig. 3 ▶ *f*). The pieces of information gathered by temperature-controlled crystallography were valuable elements for solving the complex puzzle of substrate and product traffic in one of nature’s fastest enzymes.

As seen in the aforementioned and other examples (Dub­novitsky *et al.*, 2005 ▶), protein active sites are particularly radiation-sensitive. As a consequence, careful control experiments have to be carried out in order to deconvolute radiation damage from functionally relevant features in a protein structure. This is particularly true if X-rays are used to trigger a reaction. Monitoring the exact X-ray dose is obviously mandatory in this context (Murray *et al.*, 2005 ▶). Since secondary radiation damage is temperature-dependent (§[Sec sec5]5), if the data-collection temperature is changed between data sets control experiments must accompany any crystallographic investigation using a brilliant synchrotron source.

## 7. Perspectives

Macromolecular crystallography as a function of temperature currently comprises a small niche of experiments that can be enlarged. The possibility of performing slow-cooling experiments (Warkentin & Thorne, 2009 ▶) has already been discussed in §[Sec sec4]4. Comparing protein structures determined during slow cooling and during slow heating after flash-cooling might teach us more about the ensemble of conformational substates trapped in a flash-cooled crystalline protein. Temperature-controlled crystallographic experiments could also be carried out with neutrons instead of X-rays. Neutron crystallography allows the visualization of protons, which can be of interest for the interpretation of enzymatic intermediate states. Temperature-controlled kinetic neutron crystallography will be more accessible when more open-flow cooling systems are available on neutron diffractometers. Another perspective is to multiply temperature-controlled X-ray crystallographic experiments on membrane proteins (Hirai *et al.*, 2009 ▶). To this end, carefully characterizing the temperature-dependent X-ray diffraction of membrane-protein crystals and their lipid and/or detergent matrix will be beneficial. Neutron spectroscopy experiments have indeed shown that lipid rather than water dynamics control the dynamics of membrane proteins (Wood *et al.*, 2007 ▶). There is certainly a need to further explore the temperature-dependence of X-ray radiation damage to macromolecular crystals and their components. *In crystallo* spectroscopic techniques (UV–vis, Raman, EPR, XAS *etc*.) are a valuable complement to crystallography in this context.
